# Proliferative double-negative T (DNT)-cell responses to PD-1 blockade therapy were positively correlated with good clinical outcomes in non-small cell lung cancer patients

**DOI:** 10.12669/pjms.42.1.11433

**Published:** 2026-01

**Authors:** Ying Mu, Xiaofang Wei, Yukun Wang, Weihong Sun

**Affiliations:** 1 Ying Mu Department of Clinical Laboratory, Qingdao Central Hospital, University of Health and Rehabilitation Sciences (Qingdao Central Hospital), No. 127, Siliu South Road, Qingdao 266042, China; 2 Xiaofang Wei Department of Clinical Laboratory, Qingdao Central Hospital, University of Health and Rehabilitation Sciences (Qingdao Central Hospital), No. 127, Siliu South Road, Qingdao 266042, China; 3 Yukun Wang Shandong Academy of Chinese Medicine, No.7 Yanzi West Road, Jinan of Shandong Province, 250014, China; 4 Weihong Sun Biotherapy Center, University of Health and Rehabilitation Sciences (Qingdao Central Hospital), No. 127, Siliu South Road, Qingdao 266042, China

**Keywords:** Checkpoint inhibitors, Cancer immunotherapy, Double-negative T-cells, PD-1, NSCLC

## Abstract

**Objective:**

To explore the correlation between the frequency of circulating double-negative T cells (DNT) and clinical outcomes of tumor immunotherapy in non-small cell lung cancer (NSCLC) patients.

**Methodology:**

We conducted a retrospective, single-center study at Qingdao Central Hospital, China (from October 2020 to February 2023) involving a cohort of patients with advanced-stage NSCLC who received PD-1-targeted therapies. A basal and longitudinal analysis of peripheral DNT cells was performed on this cohort. The frequency and effector phenotypes of circulating DNT cells were examined using flow cytometry . The cytotoxicity of DNTs was assessed using DELFIA EuTDA cell cytotoxicity assay kits.

**Results:**

Flow cytometry analyses showed a marked reduction in circulating DNTs in patients with late-stage (III/IV) NSCLC compared to those with early-stage (I/II) disease. Interestingly, we observed an increase in Ki-67+ DNT cells in approximately 57% (29/51) of late-stage NSCLC patients following the first cycle of anti-PD-1 treatment, and these proliferating DNT cells exhibited effector-like phenotypes and enhanced cytotoxicity towards the lung adenocarcinoma cell line A549. Notably, 70.27% (26/37) of patients who experienced clinical benefits showed a responsive DNT cell profile within four weeks of starting therapy, but not 82.35% (14/17) of patients who developed disease progression. Strikingly, patients with early proliferative DNT cell responses had a longer overall survival (OS) than non-responders. The frequency of DNTs was positively correlated with a good clinical prognosis in patients receiving anti-PD-1 therapy.

**Conclusion:**

Analysis of pre- and post-treatment DNT cells revealed that a higher DNT cell count was associated with better prognosis. Our findings suggest that peripheral DNT cells may serve as valuable biomarkers for monitoring clinical responses in NSCLC patients undergoing anti-PD-1 therapy.

## INTRODUCTION

Approximately 85% of all lung cancer patients worldwide are diagnosed with NSCLC and it is associated with higher mortality rates.^1^ Despite the availability of various EGFR-targeted tyrosine kinase inhibitors (TKIs) for NSCLC, some patients do not benefit from these targeted therapies or platinum-based chemotherapy. In recent years, the combination of anti-PD-1 therapy with other cancer treatments has provided new hope for NSCLC patients.^2^,^3^ Expressions of the PD-1 receptor on T cells and its ligand, PD-L1, have been implicated in tumor immune evasion.^4^

Abnormal expression of PD-1 is a hallmark of exhausted T cells.^5^ Consequently, the PD-1/PD-L1 blockade axis can reinvigorate these exhausted T cells and restore anti-tumor responses.^6^,^7^ Recently, several antibodies targeting the PD-L1/PD-1 pathway have been approved and shown durable antitumor responses across multiple tumor types.^8^ However, despite the success of immunotherapy targeting the PD-1 pathway, many cancer patients do not achieve clinical responses.^9^ Thus, there is an urgent need to identify predictive markers to evaluate clinical efficacy following anti-PD-1 therapy, which can also help differentiate between successful and unsuccessful treatment outcomes.

DNT cells are characterized by the expression of CD3 but the absence of CD4, CD8, and NK T cell markers.^9^ These cells represent a unique subset of regulatory T cells that play a role in maintaining immune homeostasis *in vivo*.^10^ Research has shown that DNT cells can infiltrate solid tumors and exhibit potent anti-cancer activity against various cancers, including NSCLC, pancreatic tumors, and liver cancer.^11^,^12^ DNT cells directly kill target cells via perforin and granzyme B 13 and the Fas/FasL-mediated death receptor pathway^14^ in an MHC-unrestricted manner. Regarding cytokine production, DNT cells secrete significant amounts of IFN-γ and TNF-α. IFN-γ not only has direct anti-proliferative effects but also enhances DNT cell cytotoxicity by upregulating NKG2D.^15^,^16^ In addition, TRAIL secretion further induces apoptosis.^17^ Interactions with CD4+/CD8+ T cells are complex and dualistic. DNT cells can inhibit the activation and effector functions of conventional T cells. Conversely, they can also play a synergistic role. Moreover, DNT cells can provide activation signals to CD8+ T cells and help shape CD4+ T helper responses by secreting cytokines like IFN-γ and TNF-α,^16^ thereby amplifying the overall anti-tumor immune response. This functional heterogeneity is critical to understanding their role in the tumor microenvironment. Furthermore, Fang et al.^16^ demonstrated that anti-PD-1 therapy enhances the antitumor effects of tumor-infiltrating DNT cells expressing PD-1 in patient-derived xenograft models of NSCLC. However, there is limited understanding of the long-term responses of peripheral DNT cells to anti-PD-1 therapy. Previous studies have suggested that the CD4/CD8 ratio in peripheral blood after PD-1 treatment has a predictive effect on prognosis,^18^ but research on the prognostic effect indicated by DNT cells is still limited. In this study, we conducted both baseline and longitudinal analyses of peripheral DNTs in patients with advanced NSCLC undergoing anti-PD-1 treatment, aiming to identify a marker that could predict clinical outcomes following immune checkpoint blockade.

## METHODOLOGY

This was a retrospective study conducted from October 2020 to February 2023. Fifty one patients with late-stage (stage III/IV) NSCLC undergoing chemotherapy combined with PD-1-targeted therapy as first-line treatment at Department of Internal Medicine Oncology, Qingdao Central Hospital (Qingdao, China) were enrolled. Immunohistochemical analysis from the medical records revealed that PD-L1 expression levels in all NSCLC patients were < 1%. Additionally, 47 early-stage (stage I/II) NSCLC patients who had not received any treatment and 30 healthy controls were selected from Qingdao Central Hospital for comparison. The healthy controls matched to the patient group in terms of age, sex and smoking history (smoking status and pack-years). All control subjects had no personal history of tumors and important organ diseases (such as heart, lung, liver, or kidney disorders), autoimmune diseases, or chronic infectious diseases. Additionally, all enrolled individuals had no history of acute infections, fever, or use of immunosuppressants within the four weeks prior to blood collection. Tumors were staged according to the classification of the Union for International Cancer Control (UICC) based on the pathological TNM (pTNM) stage subsets.

### Inclusion criteria:

- Age between 36 and 85 years old.
- Histologically confirmed NSCLC.
- Not receiving any anticancer treatment.
- Free of other lung diseases, such as bronchiectasis and asthma.

### Exclusion criteria:

- Congestive heart failure.
- Patients with novel coronavirus and other viral infections or human immunodeficiency virus.
- Severe pulmonary infection or sepsis.
- Important organ dysfunctions include those of the heart, kidneys, and central nervous system.
- Patients with autoimmune diseases or those receiving long-term treatment with immunomodulators.
- Patients received steroid therapy as antiemetics.

### Ethical statement:

The present study protocol was reviewed and approved by the Ethics Committee of the Medical College of Qingdao University (No. IEC-AF-081-03.1, dated: January 19, 2020). All patient information was de-identified, and patient consent was not required for this study. Patient data will not be shared with third parties.

### Blood samples:

Blood samples (2 mL) were collected in EDTA-2Na vacuum tubes before the initiation of PD-1-targeted therapy and on day 21 after each cycle of anti-PD-1 therapy (up to four cycles and at discontinuation, with at least one post-treatment sample). The samples were stored at 4°C.

### Flow cytometry:

Fresh whole blood samples were stained with the following antibodies from BD Biosciences or Beckman Coulter: anti-CD3 (FITC, UCHT1), -CD4 (APC & APC-CY7, 13B8.2), -CD8 (PerCP, PSFCI21Thy2D3), -Ki-67 (PE, B68180), -CD38 (APC-CY7, LS198-4-3), -HLA-DR (PE-CY7, Immu-357), -CD45RA (PE-CY7, ALB11), -CCR7 (APC, G043H7), -PD-1 (PE, 560795), -Bcl-2 (PE-CY7, 6C8), -Granzyme B (PE-CY7, B46038), and -Tim-3 (APC, 7D3). Erythrocytes were lysed using lysis solution. Flow cytometry data were analyzed using the FlowJo software (Tree Star).

### Statistical analysis:

Continuous variables (such as age) are expressed as medians and ranges, while categorical data, including sex, smoking status, medical history, TNM stage, study drug, and disease response, are presented as absolute values and percentages. Statistical analyses, including receiver operating characteristic (ROC) analysis of DNT cells, were performed using GraphPad Prism 8 (GraphPad Software, La Jolla, CA, USA). Differences between means were evaluated using Student’s t-test, and one-way analysis of variance (ANOVA) was used to compare multiple groups. Pearson’s correlation was used for the correlation analysis. Kaplan-Meier curves were plotted for the survival analysis of the different patient groups. A two-tailed *p* < 0.05 was considered statistically significant for all the tests.

## RESULTS

A total of 98 patients were enrolled in the study, of whom 47 had early-stage (stage I/II) and 51 had late-stage (stage III/IV) NSCLC. Immunohistochemical analysis revealed that PD-L1 expression levels in all NSCLC patients were < 1%. Additionally, 30 healthy controls matched for age and gender were included for comparison. A summary of the patients’ demographic and clinical characteristics is provided in Table-I.

**Table-I:** Demographic and clinical characteristics of NSCLC patients on this study.

Clinical characteristics	stage Ⅲ/Ⅳ (n=51)	stage Ⅰ/Ⅱ (n=47)	HC^a^ (n=30)
Median age (range)	63.5(36-85)	52(34-61)	60(40-79)
***Gender, n (%)***			
Male	42(82)	17(36)	21(70)
Female	9(18)	30(64)	9(30)
PD-L1 expression (%)^c^	< 1%	< 1%	< 1%
***Smoking status, n (%)***			
Former	26(51)	13(27)	12(40)
Current	18(36)	4(9)	8(27)
Never	7(13)	30(64)	10(33)
***Histology, n (%)***			
Adenocarcinoma	25(49)	26(55)	NA^b^
Squamous cell carcinoma	21(41)	21(45)	NA
Poorly differentiated	5(10)	0(0)	NA
Adenosquamous	0(0)	0(0)	NA
***Stage at diagnosis, n (%)***			
Ⅰ	0(0)	30(64)	NA
Ⅱ	0(0)	17(36)	NA
Ⅲ	11(22)	0(0)	NA
Ⅳ	40(78)	0(0)	NA
***Driver mutation***			
EGFR	0(0)	NA	NA
ALK	0(0)	NA	NA
***Prior chemotherapy, n (%)***			
One line	51(100)	NA	NA
Two lines	0(0)	NA	NA
Three or more lines	0(0)	NA	NA
***Therapy***			
Monotherapy	0(0)	NA	NA
Combination therapy	51(100)	NA	NA
***Study drug, n (%)***			
Sintilimab	17(33)	NA	NA
Tislelizumab	21(41)	NA	NA
Pembrolizumab	3(6)	NA	NA
Cemiplimab	8(16)	NA	NA
Toripalimab	2(4)	NA	NA
***Best disease response, n (%)***			
Partial response (PR)	20(40)	NA	NA
Stable disease (SD)	17(33)	NA	NA
Progressive disease (PD)	14(27)	NA	NA

a: healthy controls, b: not available, c:Immunohistochemical analysis.

### Decrement of DNT cells in the peripheral blood of advanced NSCLC patients:

**Fig.1:**
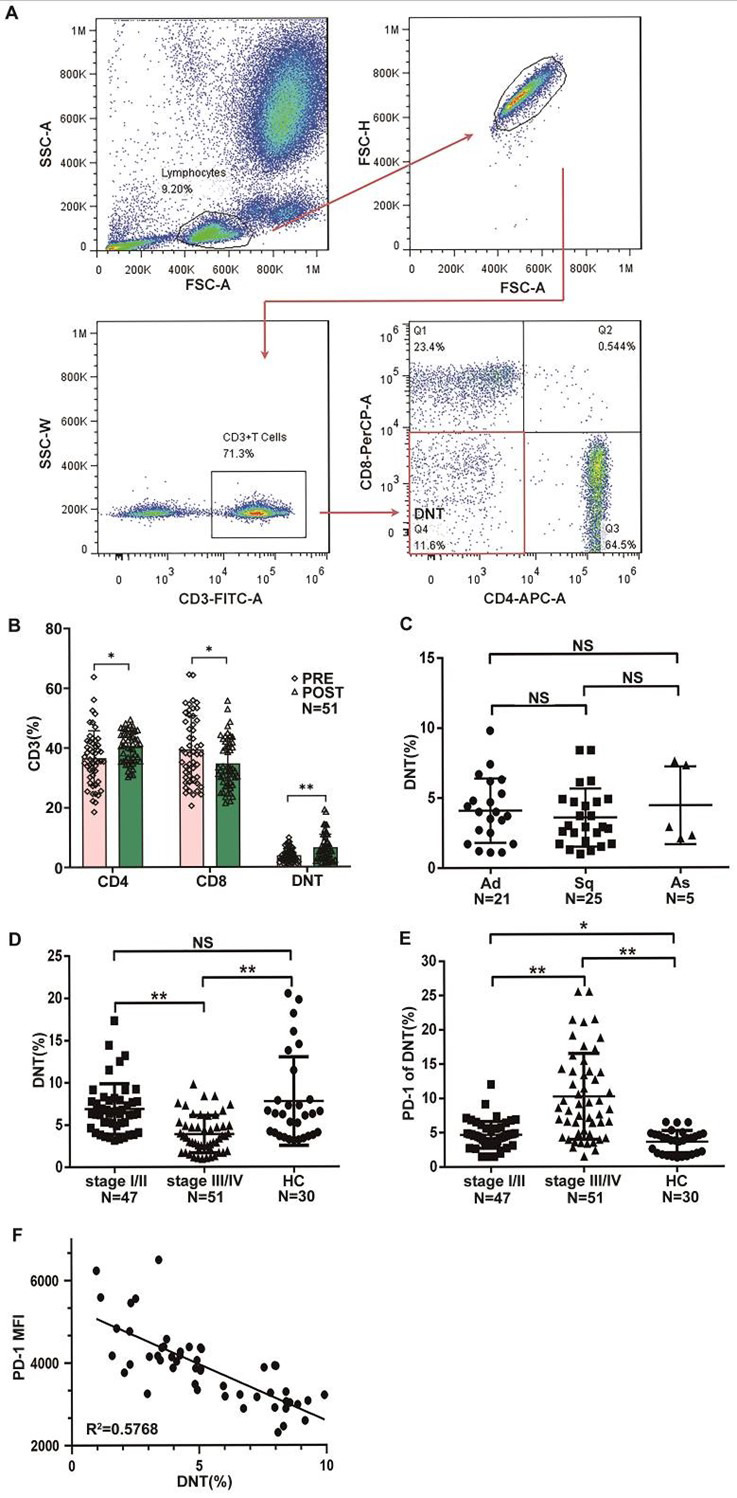
Expression of circulating DNT cells and PD-1 in NSCLC patients. Analysis was performed on whole blood obtained from NSCLC patients before the initiation of PD-1–targeted therapy. (A) Dot plots illustrate the gating strategy used to identify CD3+CD4-CD8- T cells. (B) Graph shows the proportion of T cells (CD4+, CD8+, and DNT cells) before and after anti-PD-1 therapy. Frequencies of DNT cells in patients with (C) different tumor stages and healthy controls (HC), and (D) different histological types: Adenocarcinoma (Ad), Squamous Cell Carcinoma (Sq), and Adenosquamous (As), as determined by flow cytometry (FCM). (E) Frequency of PD-1 expression on DNT cells in patients with different tumor stages and HC. (F) Correlation between PD-1 expression levels on DNT cells and the frequency of DNT cells in NSCLC patients. Each data point represents an individual subject. Data are shown as mean ± SEM; NS: Not Significant; *p < 0.05, **p < 0.01. (Early-phase I/II, n = 47; Late-phase III/IV, n = 51; Ad, n = 51; Sq, n = 42; As, n = 10; HC, n = 30).

To explore the role of peripheral lymphocytes in predicting responses of NSCLC patients to PD-1 pathway blockade, we analyzed changes in the frequency of peripheral CD4^+^ T cells, CD8^+^ T cells, and DNT cells in 51 advanced NSCLC patients before treatment and after the first cycle of anti-PD-1 therapy. Representative flow cytometry plots and gating strategies are shown in Fig.1A. We found that, compared to pre-treatment levels, the percentage of CD4^+^ T cells (PRE 36.57 ± 9.19% vs. POST 40.31 ± 5.20%, increased fold 1.10) and DNT cells (PRE 3.88 ± 2.21% vs. POST 6.47 ± 4.42%, increased fold 1.67) markedly increased after the first cycle of anti-PD-1 treatment, whereas the frequency of CD8^+^ T cells declined (PRE 39.41 ± 11.24% vs. POST 34.67 ± 8.32, decreased fold 1.14) (Fig.1B). The notable changes in the frequency of pre- and post-treatment DNT cells encouraged us to further investigate the effects of DNTs on NSCLC patients with NSCLC responses to anti-PD-1 therapy.

We examined the frequency of peripheral DNT cells in different pathological types and stage I/II and stage III/IV NSCLC patients, as well as in healthy controls, using flow cytometry. Our analysis revealed no obvious differences in the frequency of DNT cells among the different histological types of NSCLC (Fig.1C). Interestingly, there was a marked reduction in the frequency of DNT cells in stage III/IV patients (3.88 ± 2.21%) compared to stage I/II patients (6.68 ± 3.21%) and healthy controls (7.74 ± 5.01%) at baseline (before therapy initiation) (Fig.1D).

Additionally, Flow cytometry results showed that DNT cells in the stage III/IV group expressed higher levels of PD-1 than those in the stage I/II and healthy control groups (III/IV 10.30 ± 6.20% vs. I/II 5.41 ± 2.66% and healthy controls 4.60 ± 1.61 %)(Fig.1E). Correlation analysis revealed that the frequency of DNTs in advanced-stage patients was negatively correlated with PD-1 levels (Fig.1F). Collectively, the expression of DNT cells was obviously declined in patients with advanced NSCLC compared with patients in stage I/II and healthy controls, which may be potentially involved in lung cancer progression.

### Augmentation of peripheral DNT cells in advanced NSCLC patients following anti-PD-1 therapy:

**Fig.2:**
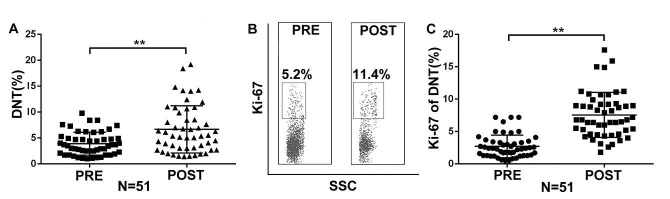
Proliferation of DNT cells in advanced-stage NSCLC patients after PD-1–targeted therapy. Analysis was performed at baseline (pre-treatment) and on day 21 after anti-PD-1 treatment initiation (post-treatment) (n = 51). Gates were determined based on CD3+CD4-CD8- T cells. (A) Graph shows the frequency of peripheral DNT cells. (B) Representative flow cytometry plots are shown. (C) Frequency of Ki-67+ cells among DNT cells (post-therapy compared with baseline). Data are presented as mean ± SEM; **p < 0.01.

To examine changes in peripheral blood DNT cells in advanced NSCLC patients undergoing anti-PD-1 checkpoint inhibition, blood samples were collected on day 21 of each treatment cycle and circulating DNT cells were analyzed by flow cytometry. We observed a significant increase in the frequency of DNT cells after the first cycle of treatment compared to that at the pre-treatment levels (PRE: 3.88 ± 2.21% vs. POST: 6.47 ± 4.42%) (Fig.2A). Ki-67 is a marker of cellular proliferation and reinvigoration. In 86.27% (44/51) of patients, the increased DNT cells following PD-1-targeted therapy were predominantly composed of Ki-67 positive cells (PRE 2.70 ± 1.73% vs. POST 7.57 ± 3.45%) (Fig.2B and 2C). These results suggest that PD-1/PD-L1 pathway blockade may stimulate the proliferation of DNT cells.

### Elevated DNT cells acquiring effector-like phenotypes after anti-PD-1 therapy:

**Fig.3:**
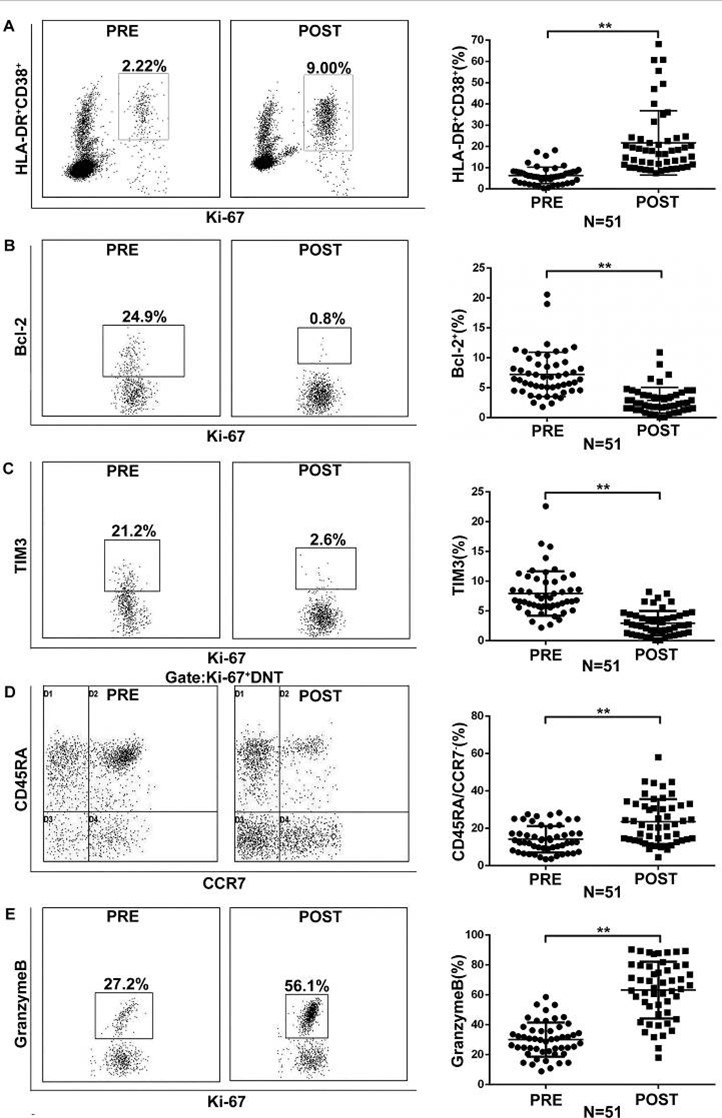
Blockade of the PD-1 pathway induces proliferation of effector-like Ki-67+ DNT cells. Analysis was performed at baseline (pre-treatment) and on day 21 after anti-PD-1 treatment initiation (post-treatment) (n = 51). Gates were determined based on CD3+CD4-CD8- T cells. Among Ki-67+ DNT cells (post-therapy compared with baseline), (A) Frequency of HLA-DR+ CD38+ cells; (B) Frequency of Bcl-2lo cells; (C) Frequency of Tim3lo cells; (D) Frequency of CCR7-CD45RA- cells; (E) Frequency of granzyme B+ cells. Representative flow cytometry plots are shown. Data are presented as mean ± SEM; **p < 0.01.

To elucidate the mechanisms by which DNT cells predict responses to PD-1-targeted immunotherapy in NSCLC patients, we characterized Ki-67^+^ DNT cells induced by PD-1 pathway blockade. The co-expression of HLA-DR and CD38 typically identifies expanded effector T cells during immune response. Flow cytometry analysis revealed that post-treatment Ki-67^+^ DNT cells exhibited higher expression of HLA-DR and CD38 (PRE 6.31 ± 3.84% vs. POST 21.85 ± 14.88%), along with decreased levels of Bcl-2 (a downregulated regulator of apoptosis in effector cells) (PRE 7.25 ± 3.68% vs. POST 2.90 ± 2.12%) and Tim-3 (a negative regulator of T cell activation) (PRE 7.93 ± 3.70% vs. POST 2.95 ± 2.04%) compared to pre-treatment (Fig.3A–3C).

Additionally, these Ki-67^+^ DNT cells demonstrated reduced levels of CD45RA and chemokine receptor 7 (CCR7) (PRE 14.14 ± 7.03% vs. POST 23.57 ± 11.85%) (Fig.3D). Furthermore, the proportion of Granzyme B^+^(a cytotoxic protein) Ki-67^+^ DNT cells was markedly elevated in post-treatment samples compared to pre-treatment samples (PRE: 30.11 ± 11.32% vs. POST: 63.20 ± 18.83%) (Fig.3E). These findings indicate that PD-1-targeted therapies promote the proliferation of effector-like DNT cells.

### Positive associations between DNT cell responses and therapy outcomes:

**Fig.4:**
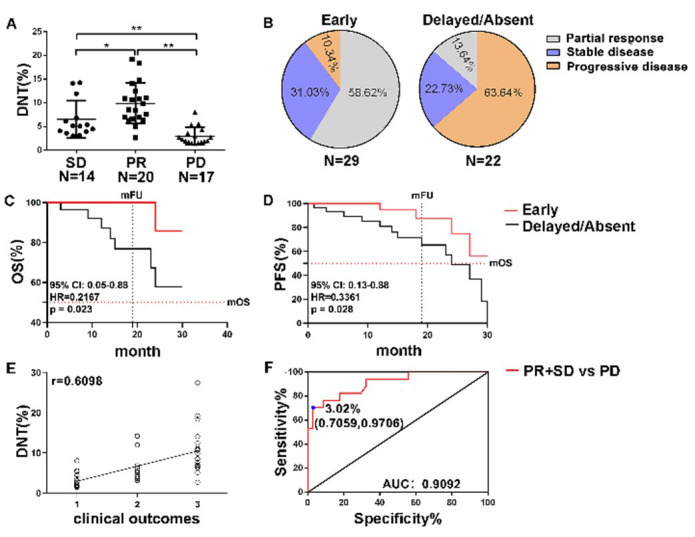
Early proliferation of Ki-67+ DNT cells correlates with clinical outcomes. Tumor response was evaluated using RECIST1.1 (n = 51). (A) Frequency of circulating DNT cells among patients with different clinical responses: partial response (PR), stable disease (SD), and progressive disease (PD). (B) Frequency of clinical outcomes in patients with early DNT-cell responses (Early) versus those with absent or delayed DNT-cell responses (Absent/Delayed). (C) Overall survival (OS) and (D) progression-free survival (PFS) curves of NSCLC patients based on the timing of DNT-cell proliferation induced by anti-PD-1 therapy: early DNT-cell responses (within the first 4 weeks after anti-PD-1 treatment initiation; Early, n = 29) versus delayed/absent DNT-cell responses (after 6 weeks post-treatment initiation; Delayed/Absent, n = 22). (E) DNT (%) were correlated with curative effect (PD=1, SD=2, PR=3) after Pearson correlation analysis (r = 0.6098, P < 0.001). (F)Receiver operating characteristic (ROC) curve for circulating DNT cells distinguishing patients with PR/SD from those with PD. OS: Overall Survival; PFS: Progression-Free Survival.

Clinical responses to anti-PD-1 immune checkpoint blockade therapy were assessed using the RECIST 1.1 criteria. As illustrated in Fig.4A, patients with a partial response (PR 21/51) exhibited the highest frequency of DNT cells, whereas those with progressive disease (PD 17/51) showed a marked decrease in DNT cells compared to patients with stable disease (SD 14/51) (PR 9.89 ± 3.94% vs. SD 6.51 ± 3.94% vs. PD 3.01 ± 1.84). Early detection (29/51) of Ki-67^+^ DNT cell responses within four weeks of initiating anti-PD-1 therapy revealed that 89.66% (26/29) of patients with good prognosis exhibited an average 1.6-fold increase in Ki-67^+^ DNT cells, while only minimal differences were observed in 63.64% (14/22) of patients with poor prognosis.

Specifically, among patients with early Ki-67^+^ DNT cell responses, 58.62% (17/29) achieved PR, 31.03% (9/29) had SD, and 10.34% (3/29) experienced PD, respectively. In contrast, among patients with poor prognosis, only 13.64% (3/22) and 22.73% (5/22) achieved PR and SD, respectively, whereas 63.64% (14/22) showed PD (Fig.4B). This data suggests that an early increase in Ki-67^+^ DNT cells following anti-PD-1 treatment is associated with better clinical outcomes. Furthermore, at the endpoint of the 30 months follow-up (median follow-up time: 19 months), one out of 29 patients with early proliferative DNT cell responses died, and four experienced disease progression. In contrast, 8 out of 22 patients with absent or delayed DNT cell responses died, and 13 developed disease progression.

In addition, patients with DNT-cell proliferation within the first four weeks after anti-PD-1 treatment initiation was defined as early responder (Early); Patients who fail to achieve a DNT cell proliferation by week six of initial treatment are classified as delayed/absent responder (Delayed/Absent). At a median of 19 months of follow-up (range, up to 30 months), overall survival (OS) and progression-free survival (PFS) analyses showed that patients with early proliferative DNT cell response exhibited higher median OS compared with patients with descent or delayed DNTs response (Undefined vs. Undefined, 95% CI: 0.05-0.88, HR=0.2167, *p* = 0.023) and median PFS compared to patients with descent or delayed response (Undefined vs. median=24 months, 95% CI: 0.13-0.88, HR=0.3361, *p* = 0.028) (Fig.4C and 4D). In addition, the frequency of DNTs was positively correlated with clinical outcomes (PD, SD, PR) after Pearson correlation analysis (r = 0.6098, *P* < 0.001) (Fig.4E).

To evaluate the potential of DNT cell counts in distinguishing between patients with PR+SD and those who developed PD, we performed a receiver operating characteristic (ROC) analysis. The area under the ROC curve (AUC) for DNT cells was 0.91 (95% CI: 0.8237–0.9947, *p* < 0.001). The optimal pre-treatment DNT cell cutoff level was 3.02%, with a sensitivity of 70.59%, specificity of 97.06%, and likelihood ratio of 24.00. The cut-off means that patients have a more favorable prognosis when the frequency of DNT cells exceeds 3.02% prior to anti-PD-1 therapy. (Fig.4F).

## DISCUSSION

In this study, we conducted a longitudinal analysis of circulating DNT cells in patients with late-stage NSCLC before and after treatment with PD-1 blockade. Our findings indicated that the number of DNT cells increased following PD-1 blockade therapy, and the proliferation of DNTs was positively correlated with clinical outcomes. Notably, these elevated DNT cells were predominantly Ki-67^+^ cells with an effector-like phenotype. Importantly, the levels of DNT cells could effectively distinguish patients with partial response (PR) and stable disease (SD) from those who developed progressive disease (PD). These results suggest that circulating DNT cells may serve as a valuable biomarker for predicting patient outcomes in immunotherapy targeting PD-1. The increase in Ki-67^+^ DNT cells following PD-1 blockade, coupled with their effector-like characteristics, underscores their potential role in monitoring and predicting therapeutic efficacy in NSCLC patients. DNT cells typically account for 1% to 5% of T cells in the peripheral blood of healthy individuals.^19^

We observed a marked decline in the baseline levels of circulating DNT cells in patients with advanced NSCLC compared to those with early-stage NSCLC and healthy controls. Strippoli et al.^20^ reported that a higher absolute number of DNT cells at baseline was associated with fewer metastatic sites and better ECOG performance status. Consistent with these results, our findings indicated that the percentage of circulating DNT cells was markedly lower in NSCLC patients with metastasis than in those with early-stage disease. However, Liang et al.^21^ found no obviously differences in the frequency of circulating DNT cells between stage I and metastatic NSCLC patients, and Strippoli et al. demonstrated the median absolute and relative value of DNTs decreased in patients who responded to checkpoint inhibitors,^20^ highlighting some discrepancies in the literature. The function of DNT cells is highly dependent on the specific pathological context. DNT cells exhibit distinct anti-tumor properties across different tumor types.^22^,^23^ The TME of melanoma may differ significantly from that of NSCLC. These differences may influence the activation status of DNT cells, their cytokine secretion (e.g., IL-10, IFN-γ, IL-17), and their interactions with other immune cells such as CD8^+^ T cells, ultimately leading to divergent roles of DNT cells. DNT cells found in the lymph nodes of melanoma patients express tolerance-associated biomarkers such as CD30, and their increased frequency correlates with disease progression, suggesting a more suppressive role in melanoma.^24^,^25^ DNT cells from different sources exhibit distinct characteristics.^23^ Fang et al. observed that peripheral blood DNT cells proliferate after anti-PD-1 therapy and are associated with a favorable prognosis.^16^ In contrast, conflicting findings in melanoma (decrease in DNT cells) may stem from differences in the DNT populations studied (e.g., circulating DNT cells vs. tumor-infiltrating DNT cells) or from the melanoma TME preferentially driving DNT cells toward a dysfunctional or exhausted state. It has been reported that tumor-infiltrating DNT lymphocytes in melanoma lymph node metastases exhibit tolerogenic features, and their increase correlates with disease progression, further supporting the tumor-type-specific functionality of DNT cells. Furthermore, differences in treatment response may exist: the same therapy (e.g., anti-PD-1) may differentially modulate DNT cells within the TME of different tumors. The response pattern of melanoma to immune checkpoint inhibitors and the subsequent remodeling of the TME may differ from that in NSCLC, potentially leading to divergent DNT cell responses to therapy. For instance, melanoma may possess stronger intrinsic immune resistance mechanisms that limit the functional recovery of DNT cells following anti-PD-1 treatment. Therefore, our findings in NSCLC are not necessarily contradictory to certain reports in melanoma. Instead, they underscore the importance of investigating DNT cell biology within specific tumor types and therapeutic contexts. Future studies are needed to further elucidate the precise molecular mechanisms that drive the functional divergence of DNT cells across different cancers.

Anti-PD-1 therapy has been shown to reinvigorate T cell activation^26^,^27^, Consistent with previous findings that anti-PD-1 therapies increase the number of circulating CD8^+^ T cells and tumor-infiltrating DNT cells in NSCLC patients^16^,^28^, our study also observed an increase in peripheral Ki-67^+^ DNT cells within four weeks of therapy initiation. However, Stripppli et al.^20^ reported a decrease in DNT cells in metastatic melanoma patients undergoing immune checkpoint inhibition, suggesting variability in the response across different cancer types.

Recent studies have demonstrated that CD8^+^ T cells activated by PD-1-targeted therapies or live attenuated viruses exhibit low levels of Bcl-2, TIM-3, CCR7, and CD45RA, alongside high levels of HLA-DR and CD38.^29^-^31^ Similarly, in our study, the responding DNT cells after anti-PD-1 therapy also exhibited these characteristics. DNT cells have emerged as potent antitumor effectors due to their high cytotoxicity against a range of tumor cells.^15^ In this study, in vitro expanded DNTs from patients with a higher frequency of DNT cells exhibited enhanced cytotoxicity against A549 and these patients demonstrated partial response (PR) and stable disease (SD) after anti-PD-1 therapy, which contrasts with findings in melanoma patients.^20^ However, Fang et al.^16^ also demonstrated that anti-PD-1 therapy enhanced DNT cell-mediated antitumor activity by increasing DNT cell infiltration into tumor sites and inhibiting late-stage lung cancer progression *in vivo*.

Our study demonstrates that PD-1 blockade promotes the proliferation and effector phenotype acquisition of circulating DNT cells in advanced NSCLC. While the precise mechanisms warrant further investigation, a framework involving direct and indirect effects can be proposed. Directly, DNT cells from patients express PD-1 at levels that were negatively correlates with their frequency, suggesting functional inhibition via the PD-1/PD-L1 axis within the TME. Anti-PD-1 antibody may directly relieve this intrinsic inhibition, enhancing TCR signaling to drive proliferation (increased Ki-67) and boost cytotoxicity (upregulated granzyme B), consistent with known TCR-dependent DNT cell activation. Indirect effects are likely predominant. PD-1 blockade reactivates APCs and reinvigorates CD8^+^ T cells, collectively reshaping the immune milieu. Reactivated APCs can provide stronger co-stimulation to DNT cells, and cytokines like IL-15 can enhance their cytotoxicity.^17^ Thus, DNT cell revival is probably part of a coordinated anti-tumor response. The functional plasticity of DNT cells is swayed by the microenvironment, which PD-1 blockade skews toward an anti-tumor state.^22^ Furthermore, combination chemotherapy may synergize. Agents like daunorubicinor azacitidine can upregulate stress ligands (e.g., for NKG2D/DNAM-1) on tumor cells via DNA damage or STING pathway activation.^15^,^32^ This may pre-sensitize tumor cells to the attack by reinvigorated DNT cells, amplifying the therapeutic effect. In conclusion, PD-1-driven DNT cell reactivation likely involves direct signal disinhibition and potentiation within a remodeled microenvironment. Future studies using single-cell sequencing and co-culture models are needed to dissect the relative contributions of these pathways.

### Limitations:

Our study had some limitations. It includes the timing of blood sampling may not have been optimal. More frequent sampling may be needed, particularly within the first month of therapy, to provide more comprehensive monitoring; The limited volume of blood samples restricted the ability to directly evaluate the cytotoxicity of DNTs against lung cancer cells without in vitro amplification; The sample size was not large enough to draw definitive conclusions. Building on this, the modest cohort size (n=51) is a key constraint. Subgroup analyses (PR, SD, PD) further reduced statistical power, increasing the risk of overinterpreting the observed outcomes.

## CONCLUSION

Our study indicates that increased peripheral DNT cell expression during PD-1-targeted therapy in NSCLC patients can serve as a valuable biomarker to monitor clinical responses. The early proliferation of DNT cells after PD-1 blockade appears to be correlated with better therapeutic outcomes. If these findings are confirmed, peripheral DNT cell analysis could enhance the accuracy of predicting the responses of lung cancer patients to anti-PD-1 therapy. Further validation with larger sample sizes and at multiple centers is necessary.
